# Diagnostics and management of tuberculosis and COVID-19 in a patient with pneumothorax (clinical case)

**DOI:** 10.1016/j.jctube.2021.100259

**Published:** 2021-07-01

**Authors:** A. Starshinova, L. Guglielmetti, O. Rzhepishevska, O. Ekaterincheva, Yu. Zinchenko, D. Kudlay

**Affiliations:** a Almazov National Medical Research Centre, Saint Petersburg, Russia; b APHP, Groupe Hospitalier Universitaire Sorbonne Université, Hôpital Pitié-Salpêtrière, Centre National de Référence des Mycobactéries et de la Résistance des Mycobactéries aux Antituberculeux, F-75013 Paris, France; c Sorbonne Université, INSERM, U1135, Centre d'Immunologie et des Maladies Infectieuses, Cimi-Paris, équipe 2, F-75013 Paris, France; d Umeå University, Umeå, Sweden; e St. Petersburg Tuberculosis Hospital No. 2, Saint Petersburg, Russia; f St. Petersburg Research Institute of Phthisiopulmonology, Russia; g I.M. Sechenov First Moscow State Medical University (Sechenov University), Moscow, Russia; h NRC Institute of Immunology FMBA of Russia, Moscow, Russia

**Keywords:** Tuberculosis, Diagnosis, SARS-CoV-2 Infection, Coronavirus, Pneumothorax

## Abstract

The spread of COVID-19 in countries with high and medium incidence of tuberculosis has led to an increased risk of COVID-19 and tuberculosis co-infection, introducing new diagnostic and therapeutic challenges for the clinician. Hereby we describe a first case where tuberculosis and COVID-19 were diagnosed concomitantly in a Russian patient with pneumothorax. We discuss the challenges associated with the diagnosis and treatment of tuberculosis during the COVID-19 pandemic.

Tuberculosis prevalence and mortality rates are likely to increase substantially during COVID-19 pandemic due the disruptions of tuberculosis services including timely diagnostics [1].

Tuberculosis and coronavirus disease 2019 (COVID-19) can present with similar symptoms such as cough, fever, shortness of breath, headache, and chest pain [2], [3], causing difficulties in the differential diagnosis of these infections. To effectively prevent the spread of COVID-19, mass testing is needed using inexpensive, valid, and non-invasive methods, one of which may be the detection of the virus in saliva samples [4].

The COVID-19/tuberculosis co-infection may further complicate managing of these diseases. The risk of developing severe COVID-19 is high among elderly persons and in the presence of comorbidities such as diabetes, cancer, cardiovascular and chronic bronchopulmonary diseases [2]. Pneumothorax is a common complication in patients with chronic bronchopulmonary diseases and it has been described in patients with COVID-19 [5]. COVID-19 mortality appears to be increased in tuberculosis patients and in particular among those aged 65 years or older [6], [7].

Here, we describe a first case of successful diagnostic of tuberculosis in an older patient from Russia with COPD, emphysema, pneumothorax and COVID-19 pneumonia.

## 1 A case-report

Patient M. (59 years old), unemployed, was admitted to an Infectious Diseases Hospital in St-Petersburg, Russia, in June 2020, with symptoms of rhinitis, dry cough, fever up to 38 °C, and shortness of breath when exercising. The patient had a medical history of ischemic heart disease, chronic obstructive pulmonary disease, bullous emphysema, and previous treatment for tuberculosis in the 1980s.

Anamnesis of the disease showed that the patient started his treatment since 5th June at home with amoxicillin and clavulanic acid, a mucolytic (ambroxol), and an antiviral drug (umifenovir) due to suspicion of COVID-19. While on treatment at home, the patient's condition was rapidly deteriorating with dyspnea at rest, leading to hospitalization. At the hospital, COVID-19 was diagnosed based on a positive PCR result on June 10th.

**Fig. 1:**
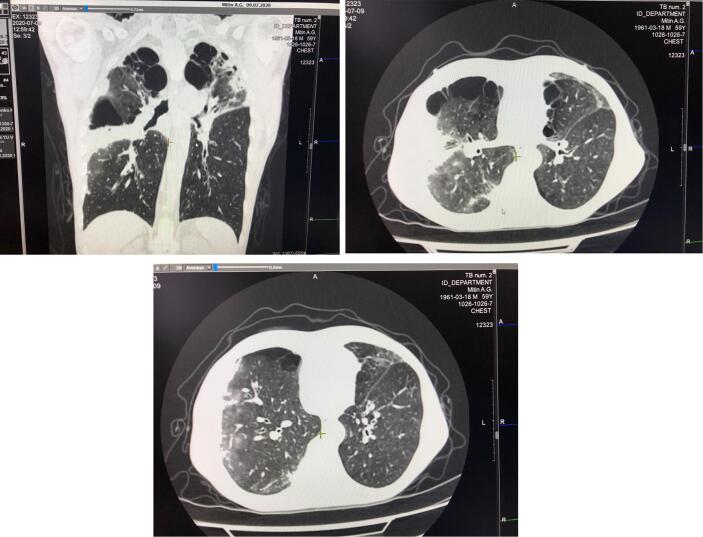
a), b), c) results of chest computed tomography scan at hospital admission.

In the hospital ward, a surgical consultation was performed and the left pleural cavity was drained with a Bülau drain, leading to a rapid clinical improvement (reduction of dyspnea). Chest Computed Tomography (CT) scan revealed signs of bilateral viral pneumonia, bullous emphysema, bronchiectasis in the upper lobes of both lungs and left-sided small pneumothorax post-drainage of the left pleural cavity; focal changes with infiltration were found in the lower lobe of the left lung (Fig. 1a–c). Paraseptal panlobular bullae were found in both lungs. The foci of ground-glass opacity and consolidations were also detected.

Based on symptoms, history and radiologic findings it was suggested that the patient developed pulmonary tuberculosis complicated by spontaneous pneumothorax occurring against the background of community-acquired bilateral pneumonia.

Acid-fast bacilli were identified in sputum by fluorescence microscopy on June 11th and 14th. A GeneXpert MTB/RIF test confirmed the presence rifampicin sensitive *Mycobacterium tuberculosis* on June 15th.

Considering the clinical data and laboratory/radiologic examinations, the patient was diagnosed with smear-positive pulmonary tuberculosis in the infiltration phase and COVID-19, complicated by spontaneous pneumothorax of the left lung.

The case management was discussed by a multidisciplinary panel, including a TB specialist, an Infectious Diseases specialist, and a thoracic surgeon. Due to severity of his condition, the patient received detoxification, broncholytic and antibiotic therapy with ceftriaxone 1 g QD, levofloxacin 100.0 BDS, and azithromycin 500 mg QD on June 10th. After the tuberculosis diagnosis was confirmed, the patient started treatment with isoniazid 600 mg QD, rifampicin 600 mg QD, ethambutol 1200 mg QD, and pyrazinamide 1500 mg QD on June 13th.

The chest drain was removed from the left pleural cavity on June 17th, with a quick recovery of the lung function. Residual subcutaneous emphysema of the postoperative wound area was observed. On June 19th and 23rd, two nucleic acid tests for SARS-CoV-2 were performed, both giving negative result and the patient was subsequently transferred to a tuberculosis hospital to continue treatment in relatively satisfactory condition. Blood test showed an increase in C-reactive protein to 65.9 mg/L, an increase in direct bilirubin (5.5 μmol/L) with no increase of total bilirubin (13.8 μmol/L), an increase in amylase (109 U/L), and a decrease in albumin (26.5 g/L) with a normal level of total protein (64.2 g/L).

The chest X-ray examination on June 30, 2020 showed improvement comparing to June 6, 2020.

The treatment was well tolerated during the entire observation period. In addition, the patient showed a clinical and radiological improvement during the treatment. However, sputum smear-microscopy and culture for *M. tuberculosis* remained positive (and drug sensitive) through July and August.

## 2 Discussion

This case demonstrates the need to exclude alternative, including infectious lung diseases in patients with suspected COVID-19 during the pandemic. A thorough study of the anamnesis and the identification of non-typical changes on the CT scan of the lungs allowed us to suspect a combination of viral pneumonia and tuberculosis in the described patient.

The influence of tuberculosis and COVID-19 on the immune status of the patient is a certain complexity, which can lead to both a more severe course of viral infection with an increase in mortality [8], but also lead to the progression or reactivation of tuberculosis, especially against the background of immunosuppressive therapy, which must be taken into account when selecting therapy [9].

So far, only a few case reports are available in literature on the co-infection of tuberculosis and COVID-19 [10], [11], [12], [13], [14], and none described a concomitant pneumothorax. The development of pneumothorax in this situation is probably due not only to the presence of bullous emphysema, but also to the development of COVID-19. The literature describes cases of spontaneous pneumothorax in these patients, even without previous mechanical ventilation, which is probably associated with the formation of structural changes in the lung tissue [5], [15], [16].

The number of cases with combined tuberculosis and COVID-19 infections will likely increase during the COVID-19 pandemic, especially in countries with a high burden of tuberculosis infection, as shown by modeling studies [17]. An additional diagnostic challenge in the management of COVID-19 is likely to occur in patients who have both active tuberculosis and HIV infection, which may influence the clinical and radiological presentation, and increase COVID-19 mortality [7], [18].

Taking into account the need for lockdown policies during the COVID-19 pandemic, different research groups have stressed the potential impact on established programs for the diagnosis and treatment of tuberculosis in different countries [1], [6], [7], [17], [19].

## 3 Conclusion

In the current epidemic situation, the global healthcare community is facing the spread of COVID-19, which exacerbates major health problems pre-existing the pandemic. Tuberculosis continues to pose a threat to lives and public health systems in many countries, due to its characteristics, which are similar to those of COVID-19: airborne transmission, predominant lung damage, the development of secondary immunosuppression and infection dissemination. COVID-19 may worsen the tuberculosis epidemic through different mechanisms: disrupting the routine process of tuberculosis detection, increasing the risk of reactivation of latent tuberculosis infection, and worsening the clinical presentation of active TB. It is therefore crucial to describe changes in the clinical presentation and morphological characteristics of tuberculosis in patients with a combination of multiple infectious diseases.

## Ethical approval

Written informed consent was obtained from the patient for publication of this case report and accompanying images. A copy of the written consent is available for review by the Editor-in-Chief of this journal on request.

## Declaration of Competing Interest

The authors declare that they have no known competing financial interests or personal relationships that could have appeared to influence the work reported in this paper.
